# Reflections on Five Decades of the Fontan Kreutzer Procedure

**DOI:** 10.3389/fped.2013.00045

**Published:** 2013-12-18

**Authors:** Christián Kreutzer, Jacqueline Kreutzer, Guillermo O. Kreutzer

**Affiliations:** ^1^Congenital Heart Surgery, Posadas National Hospital and Austral University Hospital, Buenos Aires, Argentina; ^2^Cardiac Catheterization Laboratories, Pittsburgh Children’s Hospital, Pittsburgh, PA, USA; ^3^Pediatric Cardiac Surgery, Bazterrica Clinic, Buenos Aires, Argentina

**Keywords:** Fontan Kreutzer, univentricular hearts, heart disease, cardiac surgery, atriopulmonary anastomosis, APA

## Abstract

The first successful total right heart bypass via atriopulmonary anastomosis (APA) were reported in 1971 for patients with tricuspid atresia. At the Children’s Hospital of Buenos Aires, the cohort of such procedures started in July, when the first fenestrated right heart by pass was performed, with the interposition of a homograft between the right atrial appendage and the main pulmonary artery. In the second patient, instead of placing a homograft, the APA was achieved with the patient’s own pulmonary root harvested from the outflow tract of the right ventricle. These techniques were soon replaced in 1978 with the development of the direct valveless posterior APA. Since the very beginning the principle was that the right atrium only functions as a pathway rather than a pump (reason why no inferior vena cava valves were ever used), and the diastolic properties of the systemic ventricle regulate the only real “pump” of this system. The late hemodynamic problems inherent of the APA diminished with modern surgical techniques like the lateral tunnel (LT) or the extracardiac conduit (EC). In spite of the improvement in prognosis and quality of life that the modern techniques have brought for univentricular hearts (UH), with the passing of time, deterioration of this system is frequently seen, due to chronic low cardiac output, elevated central venous pressure making heart transplantation the final stage of treatment. Progressive increase in pulmonary vascular resistances and ventricular dysfunction result in a decline in quality of life and survival. However, the timing of this occurrence is variable, and many survivors enjoy today a satisfactory clinical status. The challenge is to develop a better solution for UH, but in the mean time the Fontan Kreutzer palliation represents the best and only surgical option. It is undoubtedly one of the triumphs of cardiac surgery in congenital heart disease.

## History of the First Atriopulmonary Connection

Innovation often results from an unsolved need, as the application of new solutions to meet new requirements. In the early 70s, our field achieved excellent survival for the most complex forms of biventricular hearts by many centers in the world. However, patients with univentricular hearts (UH) were typically palliated with systemic to pulmonary shunts, pulmonary artery bandings, and partial right heart by pass such as the classic Glenn shunts and other variants (1–3). Therefore a hemodynamic “solution” for UH that was already suggested (4) was required.

**Figure 1:**
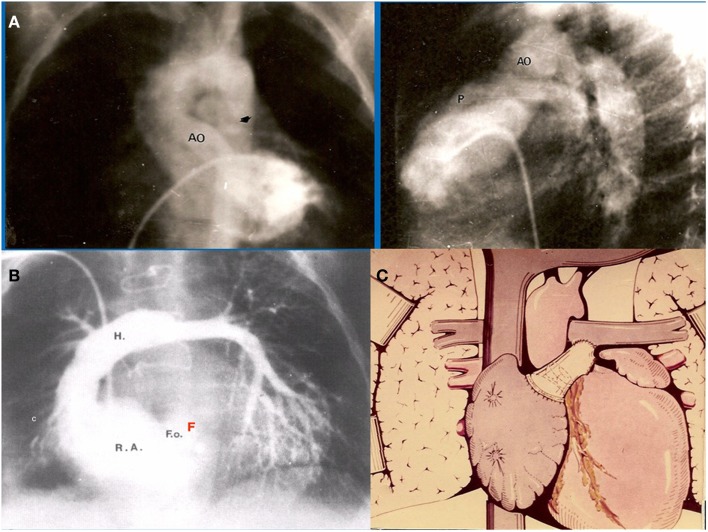
**(A)** Preoperative angiogram of our first patient showing occluded right pulmonary artery. Only small flow is seen into the left pulmonary artery **(B)** Postoperative angiogram demonstrates widely patent APA with interposition of a homograft. Note there is a small atrial communication left open, acting as a fenestration (F). **(C)** Schematic representation of the original APA performed in this patient, communicated in J. Thorac. Cardiovasc. Surg.

The first atriopulmonary anastomosis (APA) was performed without awareness of the pioneer work done by Professor Fontan, published in January 1971 (5). History shows multiple examples of independent discoveries of the same scientific idea or invention (6). Considerable work on multiple simultaneous discoveries has been achieved by William Ogburn and Dorothy Thomas. They established a list of 148 independently duplicated scientific and technological discoveries. They suggested these discoveries became virtually inevitable as knowledge accumulated within any given society and the needs of that society caused attention to be directed toward problems associated with meeting those needs. Similarly, the need to find a solution for single ventricle physiology was clearly there in the early 70s. In July of that same year (1971), a moribund, severely cyanosed 3-year-old boy with tricuspid atresia Ib, was admitted to the cardiology ward at the Ricardo Gutierrez Children’s Hospital. He had severe systemic desaturation due to a thrombosed right pulmonary artery due to a previous Waterston shunt (Figure 1A). Contrary to Professor Fontan’s first patient for whom two traditional surgical alternatives would have been available (a Blalock–Taussig Shunt or a Glenn procedure), the only theoretical options for this patient were both innovative surgical procedures: the APA (6) or enlargement of the ventricular septal defect (7). It was decided to perform an APA, placing a homograft between the right atrial appendage and the main pulmonary artery without doing a Glenn procedure, without valve implantation in the inferior vena cava. A 6 mm fenestration at the atrial septum was deliberately left open. This first patient was presented as a case report in August 1971 at a meeting of the Argentinean Society of Cardiology (6) (Figure 1B). Certainly, this was the first fenestrated total right heart by-pass ever performed in the world (8), as stated in the original article: “The total or partial closure of the foramen ovale is a subject of discussion. Although its partial closure provides a safety valve for the right atrium it also may cause a certain degree of systemic desaturation” (Figure 1C).

**Figure 2:**
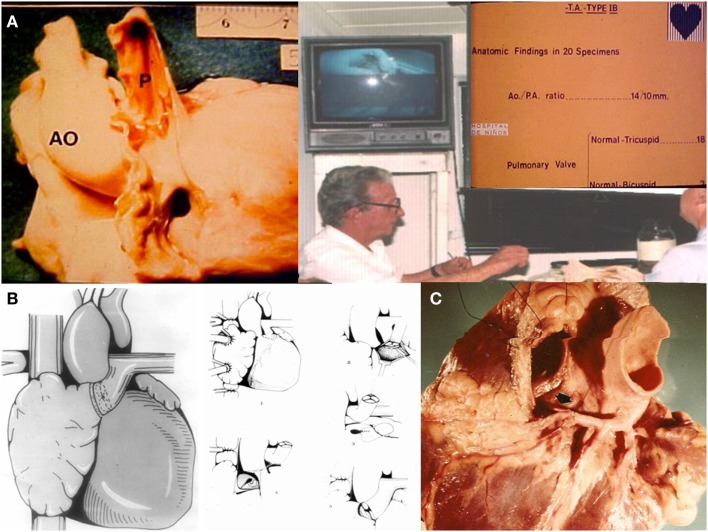
**(A)** Morphologist Dr. Luis Becú showing that the pulmonary artery is usually normal in TA Ib. **(B)** APA with the patient own pulmonary valve removed from the outflow tract of the right ventricle. **(C)** Mr. Donald Ross’s technique for the removal of the pulmonary annulus.

Dr. Luis Becú (Figure 2A) who was a superb cardiac morphologist, noticed that in tricuspid atresia Ib the pulmonary valve is most commonly anatomically normal. Therefore, in December 1971 in another patient with the same condition, it was decided to perform the APA directly (Figure 2B) with the patient’s own pulmonary root and valve (8), speculating on the possibility of growth and prevention of calcification, following Mr. Ross’s guidelines (Figure 2C) on how to remove the pulmonary annulus from the outflow tract of the right ventricle.

Discussions with Drs. Luis Becú and Alberto Rodríguez Coronel (Head of the Cardiac Catheterization Laboratory) on those days were about the following dilemma: is the right atrium functioning as a pump as suggested by Professors Francis Fontan and Viking Bjork (9) or is it just a pathway? The conclusion that the right atrium lacked the tissue properties of a ventricular chamber and therefore would never function as a pump was soon achieved. Furthermore, Dr Rodriguez Coronel emphasized the important role of the end diastolic properties of the main ventricle, acting as a suction pump and therefore being the only pump of the total right heart by pass system. Thus, the right atrium would only function as a pathway and would never do so as a ventricular pump.

Sir Magdi Yacoub, in London expanded the application of the procedure to double outlet left ventricle, reporting in 1975 with a homograft with valves in the superior vena cava and inferior vena cava. Of interest, he performed in one of the patients of his series presenting with subaortic stenosis what we wrongfully call nowadays a Damus–Kaye–Stansel (a pulmonary to ascending aorta anastomosis) procedure for single ventricle and aortic obstruction (10, 11). This concept was later re appraised by Dr. William Norwood (12).

The initial experience with the APA was presented at the centennial celebration of the Toronto Sick Children’s Hospital in 1975 (13). It was emphasized the importance of “sinus rhythm to reduce left atrial pressure to obtain a reasonable gradient (about 6 mmHg) between the right and left atrium,” and “the left heart being normal.” Regarding the need for valves at the inferior vena cava and “outlet” of right atrium it was stated that: “because of the continuous venous flow we doubt that those valves would work adequately and they could increase peripheral venous pressure and the subsequent edema.” In this regard, Dr. Rodriguez Coronel acknowledged that the pulmonary valve would be open in the whole cardiac cycle, and only would close in the event of Valsalva o coughing.

**Figure 3:**
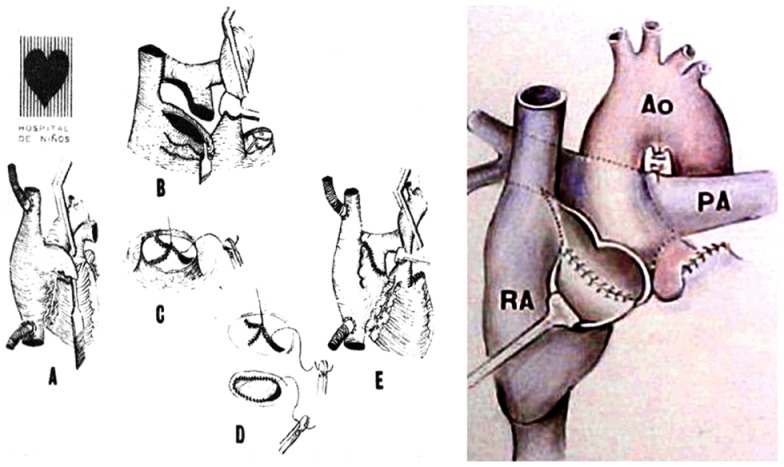
**Illustrations demonstrate the steps of the technique used for the posterior APA developed in 1978 presented in London, 1980**.

**Figure 4:**
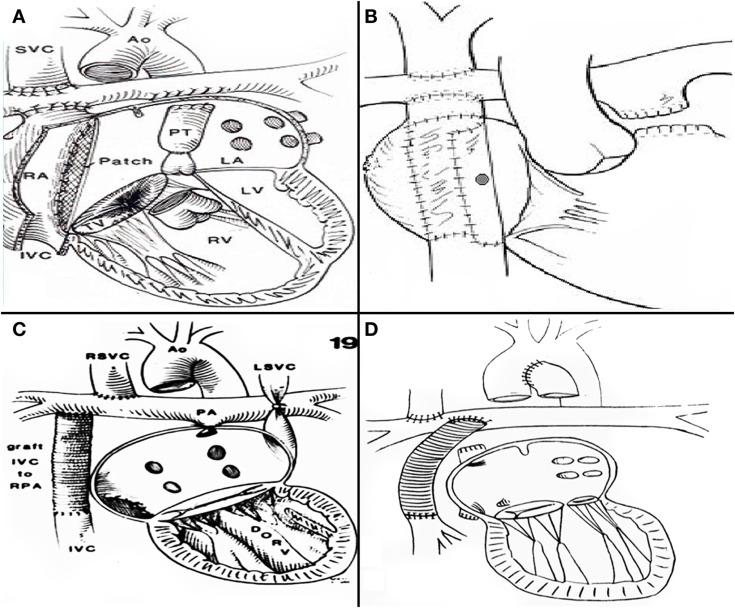
**(A)** De Leval’s total cavopulmonary connection **(B)** fenestrated lateral tunnel technique. **(C)** Puga’s extracardiac conduit technique **(D)** Marcelleti’s extracardiac conduit technique.

Finally, in 1978 it was clear that the interposition of a pulmonary valve was deleterious in this system, even more if associated to obstruction (frequently seen in homografts placed in the right side of the heart). Therefore, the surgical approach was completely changed: the concept of the largest posterior APA without a valve was introduced, moving the pulmonary trunk to the right, posteriorly and behind the aorta (Figure 3), avoiding the possibility of sternal compression. This new approach was presented in 1980 in London (14) at World Congress of Pediatric Cardiology, and published (Figure 4) in several journals (12, 15, 16).

## Norwood’s Revolution and Development of Improved Surgical Techniques

In the early 80s Dr. William Norwood at Children’s Hospital of Boston and Philadelphia was confronting the biggest challenge in congenital heart surgery: an operation for hypoplastic left heart syndrome (17), a condition that was uniformly lethal at the time. Not only Dr Norwood envisioned the procedure that allowed survival for such complex lesion, he also devised a staged pathway of treatment for all forms of UH. Depending on the initial clinical presentation a neonatal palliation, with a Modified Blalock–Taussig Shunt (MBTS), a Norwood procedure or pulmonary artery banding, as a first stage, a bidirectional Glenn or hemiFontan as a second stage in infancy, and the total right heart by pass at 2/5 years of age as a third stage.

The increasing survival of patients with single ventricle and mitral atresia or stenosis, made the atriopulmonary connections inadequate for such patients, since the need for complex atrial septations is mandatory in such scenario. Therefore, when years later, in 1988, Marc de Leval at Great Ormond Street Hospital in London, introduced the accurate concept of energy loss in a passive flow system within a large right atrium (18, 19) and described the total cavopulmonary connection (Figure 4A) his technique was widely adopted. Aldo Castañeda with Dr. Lock and Dr. Bridges re introduced the concept of a fenestration as part of the procedure (20) (Figure 4B). Prior to this, in order to avoid complex atrial septation a similar technique had been performed by Dr. Francisco Puga (21) and afterward, Drs Puga (22) and Marcelletti (23) developed the extracardiac conduit technique (EC) (Figures 4C,D), which is since 1997 most surgeons technique of choice for all patients. It can be considered controversial to determine whether the lateral tunnel (LT) or the EC is the best approach. Both techniques have excellent outcomes (20). The approach is often dependent on the preference of the cardiothoracic surgeon and team. It can also be a source of discussion whether a bidirectional Glenn or a hemiFontan is the best option.

Some differences should be pointed out between the staged LT after a bidirectional Glenn versus a hemi-Fontan. When the LT procedure is performed following a bidirectional Glenn preservation of the sinus node and its artery is jeopardized (24). LT is more frequently used in smallest patients commonly at the final stage of HLHS palliation (22, 23). However a weight less than 15 kg do not preclude the performance of an EC. With the aid of deep hypothermic circulatory arrest for the inferior anastomosis a large conduit of 16–18 mm can be easily placed in a 1-year-old infant.

The EC has a favorable balance in “bigger” patients considering the following advantages: (1) no intraatrial sutures (2) possibility of performing EC without aortic cross clamp (3) the sinus node and the crista terminalis area remains at low pressure and without suture lines in the area, avoiding injury and subsequent arrhythmias (23) (4) possibility of closing the fenestration without using a device (25). (5) The EC is a cylinder (18–20 mm) with similar inlet and outlet diameter, different than the LT that it is a truncated cone which its base (the inferior vena cava is larger than the outlet (the superior vena cava). For this reason, fenestration is more necessary with its consequent device closure (26). The presence of bilateral superior vena cava makes the mismatch more evident. In this situation, the EC should be mandatory.

**Figure 5:**
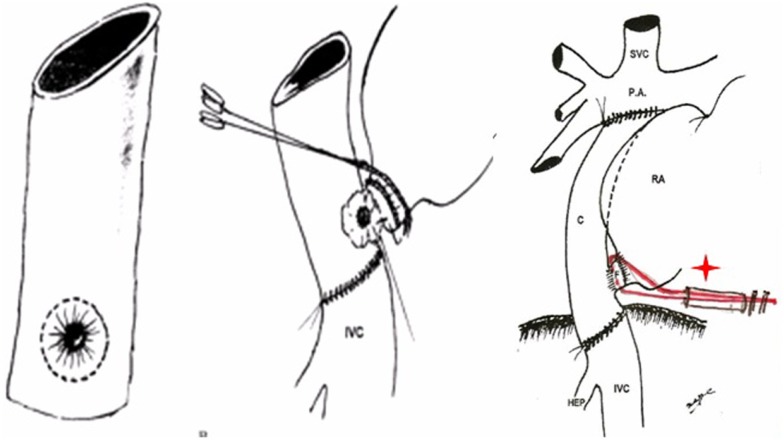
Fenestration with the skirt’s technique with snare* for deferred closing.

When a hemi-Fontan (27) or a staged LT are performed as the superior vena cava is augmented with a pericardial patch, there is higher risk of injury to the sinus node artery, given its peculiar anatomy (28). It can course in the anterior aspect of the superior vena cava, or on the posterior one or all around it, encircling it. On the other hand, the prosthetic tube of the EC does not increase its diameter (problem for “small” patients), and finally it requires extensive dissection and may induce phrenic nerve palsy (29). The latter may be avoided by using total circulatory arrest, without cannulation of caval veins. The risk of early thromboembolism could be diminished avoiding central venous lines (29). The use of a fenestrated EC with the skirt’s technique for fenestration (Figure 5) (25) allows its closure before hospital discharge according to the patient hemodynamics, by adjusting the snare under local anesthesia, without needing a device.

## Evolution of the Suboptimal Fontan Kreutzer Hemodynamic System

The natural history of the univentricular heart without surgery and with palliative procedures as shunts or bandings is disastrous. In spite of chronically limited cardiac output (30), the Fontan Kreutzer procedure has improved significantly (31, 32) the prognosis and quality of life. However, to live with only one ventricle has some limitations as a consequence of high venous pressure in the limit of the peripheral edema and limited cardiac output. The FK palliation represents without any doubt, the best surgical approach that we could offer today for these lesions. With the modern techniques, the late quality of life is remarkable, with almost 75% of survivors in NYHA class 1, in addition to an exercise tolerance of more than 60% of predicted value (31, 32).

In this matter it is quite remarkable that the Fontan Kreutzer procedure is the only palliation that provides the univentricular heart male patient the possibility of having a sexual life, since as a general rule, only a male adult who completes the first two stages of the Bruce protocol has a functional capacity >7 METs, which is considered sufficient for sexual intercourse (33). The Fontan Kreutzer procedure provides the vast majority of young adults with UH a functional capacity of more than seven METs with a mean and median close to 10 (30). On the contrary, a bidirectional Glenn with accessory pulmonary blood flow suggested as a definitive palliation by Professors Vohué and Sidi fails to provide a an exercise capacity of more than six METs in most patients (34).

Pregnancy for patients with a Fontan circulation is more likely to face obstetrical, rather than cardiovascular complications (although a decline in the cardiovascular status is frequently seen in the third trimester), including preterm labor, intrauterine growth restriction, an increased risk of cesarean section, and the potential need for anticoagulation (35).

Of note, we should be very cautious to refer to the classic articles (36) on late follow up after Fontan Kreutzer procedures performed prior 1990, and use these to predict late outcome of our current patients. These represent the results of surgical strategies and procedures that are no longer in use (i.e., long standing pulmonary artery bandings, classic right or left Blalock–Taussig shunts, late intervention, late diagnosis of significant hemodynamic problems, and inadequate surgical techniques such us the classic Fontan and Kreutzer original operations) (Table 1).

**Table 1:** **Factors that jeopardize the late outcome after a Fontan Kreutzer procedure**.

Suboptimal surgical approach
Classic Fontan
Kreutzer APA anastomosis and its variants
Bjork atrioventricular connections
History of prior long standing PA banding or shunts
Increases in pulmonary vascular resistances secondary to
Systemic atrioventricular valve disease (specially history of mitral atresia or stenosis with restrictive-interatrial-communication)
Down’s syndrome
Long duration of prior systemic PA shunts or PA distortion
Living at high altitude (54, 55)
Detrimental effects of chronic use of amiodarone
Subclinical chronic micropulmonary embolism (36)
Pulmonary lymphatic edema (due to increased central venous pressure) (56)
Chronic lack of pulsatile pulmonary flow (57).
Ventricular dysfunction
Prior long standing volume overload
Inadequate systemic ventricle (right or undetermined ventricle, cardiomyopathy, or myocardial fibrosis according to the length of time with chronic hypoxia)
Arrhythmia
AVV incompetence (more frequent in heterotaxy syndrome or HLHS)
Inadequate myocardial protection during prior operations
History of prior systemic out flow tract or arch obstructions treated with PA banding

But secondary to a multifactorial phenomenon (Table 1) it is evident that a progressive decline in functional status appears in many the FK survivors, who develop heart failure (30), arrhythmias (37), thromboembolism (38), protein losing enteropathy (39), plastic bronchitis (40), unexplained sudden deaths (41), and hepatic failure (42, 43). The reality is that these problems occur as patients do live to late follow up. Under the best circumstances using the modern surgical techniques, at least 80% of patients with single ventricle are alive after 20 years, following at least three cardiac procedures (32).

For the late survivor with “old fashioned” Fontan Kreutzer connections a conversion to LT or EC with concomitant arrhythmia surgery is indicated only for symptomatic patients with several episodes of arrhythmia in spite of amiodarone treatment or thrombus in the right atrium following Mavroudis’s guidelines (44). In failing APA the central tunnel technique may be considered as well (45). However, when ventricular dysfunction is the main determinant of the failure patients should be referred for heart transplant evaluation. Although the results of transplantation in failing single ventricle patients are not optimal, it represents the last viable option for these patients and the final stage of treatment for these patients (46, 47) and specially for HLHS. Many concerning associated problems such as renal and hepatic dysfunction can play a significant role in the outcomes. A progressive increase of pulmonary vascular resistances after transplantation for the failing Fontan palliation has also been reported (48).

Of note, there are not many patients who have reached over 50 years of age with a univentricular heart following Fontan Kreutzer palliation. To have reached 50 years of age in 2013 means that the patient was born before 1963, survived the initial palliative procedures and the original Fontan Kreutzer palliation. Of the initial cohort of Buenos Aires among the 14 patients operated upon by the early 70s, 5 are currently alive. All five with a dominant left ventricle: four of these patients had tricuspid atresia and one double inlet left ventricle. Both patients remain in good clinical condition without conversion.

**Figure 6:**
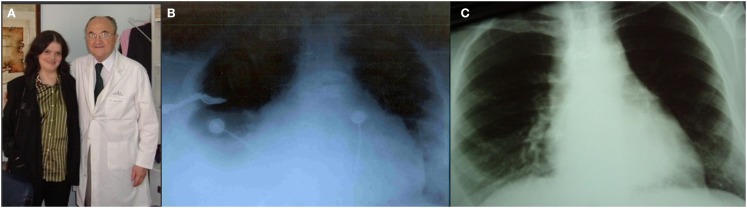
**(A)** Patient aged 53 years with TA Ib. After 34 years of Fontan Kreutzer palliation. **(B)** Pre-reconversion chest Roentgenogram. **(C)** Chest Roentgenogram 1 year after conversion.

One of the survivors, who was the fifth case of the series presented at Toronto in 1975 (13), is a lady 56 years old (Figure 6A) with TA Ib, who underwent an APA in 1975 at 17 years of age. She is currently, to our knowledge the longest survivor in the world after 39 years of FK palliation. In November 2007, her case was published as a brief communication (49).

**Figure 7:**
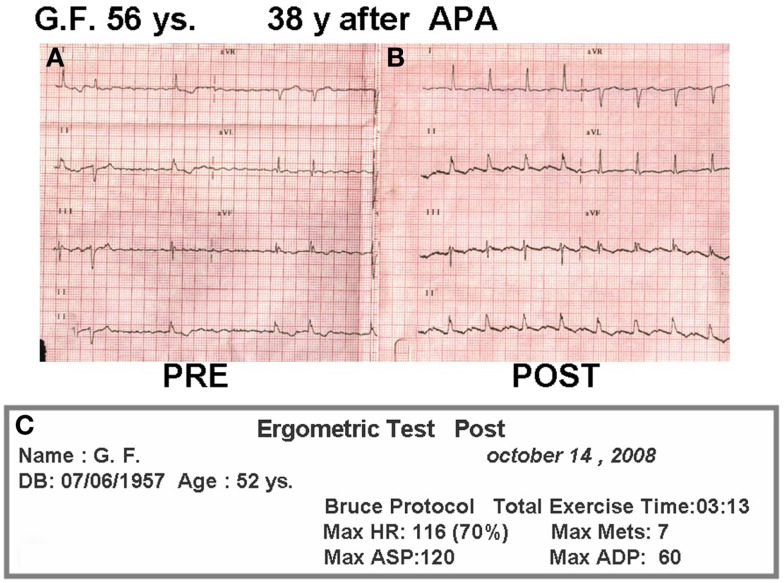
**(A)** Pre-reconversion ECG. **(B)** ECG 2 years after conversion. **(C)** Treadmill test 2 years after conversion.

For 20 years after surgery this patient led a normal life. She got married, had no pregnancies, and divorced. In the late follow-up she developed intermittent atrial fibrillation and the homograft became stenotic and calcified. She was treated with amiodarone and anticoagulation, as she originally refused surgical conversion. At age 49, she was overtly symptomatic, with hydrothorax (Figure 6B), atrial fibrillation (Figure 7A), and a large intra atrial thrombus. Fortunately her left ventricular function was preserved. Conversion to a fenestrated EC with extraction of homograft and thrombus was performed on May 2006. Now, after 7 years following conversion, she is in good clinical condition (Figure 6C) in sinus rhythm (Figure 7B) and with an exercise test achieving seven METs (Figure 7C).

## Conclusion

The durability of the suboptimal Fontan Kreutzer hemodynamic system is affected by many variables, patient, procedural, and management related. Several hypotheses could explain the deterioration of this system with the passing of time, particularly in patients with forms other than the “perfect” tricuspid atresia. Several adverse factors may lead to chronic gradual increase in the right side venous pressure: Table 1.

With the development of better surgical management, the outcome of the FK procedure seems to have improved over the years. The current long-term results using the modern surgical alternatives are excellent (31, 32, 50–52). The challenge is to reproduce this outcome for the vast majority of patients, and to develop viable alternatives to deal with late failure.

As physicians involved in the care of the most complex form of congenital heart disease, our future goal would be to seek a better surgical procedure to improve the long-term outcome of patients with UH. May it be a complete or partial mechanical device (53) or improved heart transplantation availability and outcomes. In the meantime, the Fontan Kreutzer palliation, which seemed to be a radical approach in the 70s, has become a routine procedure world wide, and continues to represent the best surgical option we can offer to these patients. It is clearly, one of the triumphs of cardiac surgery in congenital heart disease.

## Conflict of Interest Statement

The authors declare that the research was conducted in the absence of any commercial or financial relationships that could be construed as a potential conflict of interest.
